# Clinical Efficacy and Nephrotoxicity of Colistin Alone versus Colistin Plus Vancomycin in Critically Ill Patients Infected with Carbapenem-Resistant *Acinetobacter baumannii*: A Propensity Score-Matched Analysis

**DOI:** 10.3390/pharmaceutics13020162

**Published:** 2021-01-26

**Authors:** Wasan Katip, Peninnah Oberdorfer

**Affiliations:** 1 Department of Pharmaceutical Care, Faculty of Pharmacy, Chiang Mai University, Chiang Mai 50200, Thailand; 2 Epidemiology Research Group of Infectious Disease (ERGID), Chiang Mai University, Chiang Mai 50200, Thailand; oberdorferp@gmail.com; 3 Division of Infectious Diseases, Department of Pediatrics, Faculty of Medicine, Chiang Mai University, Chiang Mai 50200, Thailand

**Keywords:** colistin, vancomycin, combination, multidrug-resistant *Acinetobacter baumannii*

## Abstract

*Acinetobacter baumannii* has emerged as a significant concern worldwide. The mortality rate of carbapenem-resistant *A. baumannii* (CRAB) is increasing, especially in the intensive care unit (ICU). Thus, the objective of this study is to compare the efficacy and safety of colistin plus vancomycin for the treatment of critically ill patients with CRAB in Chiang Mai University Hospital. We conducted a retrospective cohort study of critically ill patients in the ICU with CRAB infection who received colistin alone or colistin-vancomycin combination therapy at Chiang Mai University Hospital. A total of 365 critically ill patients met the inclusion criteria. The results in this study showed that after propensity score matching, colistin plus vancomycin showed no significant differences in the 30-day mortality compared to colistin alone. Likewise, for colistin plus vancomycin, compared with colistin therapy alone, there were no significant differences in the clinical response, microbiological response and nephrotoxicity. In conclusion, colistin plus vancomycin was no significant differences in 30-day mortality, clinical response, microbiological response compared to colistin alone for infections due to CRAB. The nephrotoxicity rates were similar for both groups, so colistin combination with vancomycin was not necessary for the management of infection caused by CRAB.

## 1. Introduction

Carbapenem-resistant *Acinetobacter baumannii* (CRAB) has emerged as a significant concern worldwide. In Thailand, CRAB is now one of the most common nosocomial pathogens, especially in the intensive care unit (ICU) [1]. Moreover, CRAB infections have demonstrated increased lengths of hospital stays, mechanical ventilation and mortality ranging from 17 to 66% [2] because of critically ill patients whose prognosis is typically influenced by CRAB infection [3]. Thus, appropriate treatment regimens for effective treatment of CRAB infections are very important and should be of concern [4].

Colistin is a salvage therapy for nosocomial infections caused by CRAB in the ICU [5]. However, an important limitation of colistin is a proclivity to the emergence of heterogeneous colistin resistance during treatment, especially if used as monotherapy [6,7]. Furthermore, the emergence of CRAB adds to perennial questions surrounding colistin: whether their use in combination with other antibiotics results in enhanced activity against colistin susceptible bacteria and whether this leads to improved clinical outcomes in difficult-to-treat infections caused by CRAB infection. In vitro studies demonstrate the synergistic killing of *A. baumannii* when colistin is paired with vancomycin, a carbapenem, or rifampicin [8]. Vancomycin, as a glycopeptide, is an antibacterial which acts by inhibiting bacterial peptidoglycan synthesis and has been used to treat methicillin-resistant *staphylococcus aureus* (MRSA). Vancomycin is widely prescribed in hospitals, especially in critical care settings [9].

Novel combinations of colistin plus vancomycin have recently been claimed to work in synergy against CRAB, resulting in rapid bactericidal activity in time–kill curves [10]. Although the combination of vancomycin and colistin has demonstrated promising in vitro and in vivo results, there are concerns regarding its clinical application. Moreover, colistin is commonly used in critically ill patients for the treatment of multidrug-resistant (MDR) Gram-negative bacterial infections [3,4]. Vancomycin has been commonly co-administered with colistin and could increase the risk of colistin nephrotoxicity, but this is still not proven [11]. However, to date there have been few clinical studies directly evaluating the efficacy and safety of colistin plus vancomycin in combination in critically ill patients. We therefore aimed to compare the efficacy and safety of colistin plus vancomycin for the treatment of critically ill patients with CRAB.

## 2. Materials and Methods

A retrospective cohort study was conducted at Chiang Mai University Hospital (CMUH), a tertiary care teaching hospital in Chiang Mai, Thailand, from January 2010 to August 2017. This study was approved by the ethics committee on human research of the Faculty of Medicine (Study code: NONE-2560-04839, approved: 31 October 2017), Chiang Mai University with a waiver of informed consent for retrospective data collection under the condition of anonymously stored data collected. All methods were performed in accordance with the relevant guidelines and regulations. The criteria used to identify and classify infections were those of the Center for Disease Control and Prevention (CDC) [12], according to infectious disease (ID) physicians’ evaluations. All critically ill patients aged 18 or older admitted to the ICU who had culture positive for CRAB and clinical signs consistent with infection, who received colistin for more than 2 days to treat the documented CRAB infection, were included. Only the first episode from each patient was considered. Patients with CRAB cultures assessed to be colonizers or contaminants or who had incomplete patient records were excluded. The patients were divided into two groups: colistin alone versus colistin plus vancomycin. Patients who received colistin alone were administered an intravenous (i.v.) loading dose of 300 mg (9 million units of colistimethate sodium) of colistin base activity (CBA) followed by 150 mg CBA i.v. every 12 h (corrected according to renal function) and classified as the colistin group. Patients who received vancomycin from the onset of colistin, with both antimicrobials coinciding for at least 3 days, were termed the colistin plus vancomycin group and received colistin at a loading dose of 300 mg CBA followed by 150 mg CBA i.v. every 12 h.

Vancomycin was administered in a 60 min i.v. infusion (2 g/day in patients with normal renal function). Doses were adjusted in the case of renal dysfunction, following the manufacturer’s recommendations.

### 2.1. Data Collection

Data from the patients’ medical records and from hospital computerized databases were recorded. Data collected included age, sex, duration of antibiotic treatment, underlying disease (hypertension, cardiovascular disease, diabetes mellitus, chronic kidney disease, chronic obstructive pulmonary disease, malignancy, chronic liver disease), septic shock, mechanical ventilation, Charlson score, length of hospital stay, APACHE II score, baseline SCr, baseline glomerular filtration rate (GFR), total colistin dose, type of nephrotoxic medications, sources of CRAB infection, mortality status.

### 2.2. Outcomes Measurement

Efficacy was assessed based on 30-day mortality, clinical responses and bacteriological responses to the therapy. The primary outcome measure was 30-day mortality, defined as death within 30 days after start of colistin treatment. Secondary outcomes of concern included clinical response to therapy that was measured by resolving or partially resolving signs and symptoms of CRAB infection at the end of treatment with colistin. Clinical failure has been described as failure to fulfill all clinical response requirements during treatment with colistin. Microbiological response at the end of therapy, defined as a follow-up of two consecutive CRAB-negative cultures of clinical samples collected from the infection site after the initial positive culture, while microbiological failure was defined as the persistence of CRAB in the subsequent specimen cultures. Clinical signs and symptoms and laboratory results were evaluated as safety data. Nephrotoxicity was defined according to the risk, injury, failure, loss and end-stage kidney disease (RIFLE) criteria [13]. Evidence of nephrotoxicity from colistin was obtained from the review of physicians’ notes. Nephrotoxicity was counted if patients developed any grade of renal failure based on the RIFLE criteria.

### 2.3. Antimicrobial Susceptibility Test

Using traditional cultures and biochemical techniques, *A. baumannii* was detected at the Clinical Microbiology division, Chiang Mai University Hospital. According to the Clinical and Laboratory Standards Institute (CLSI) protocol [14], antimicrobial susceptibility to colistin has been interpreted. Identifying isolates at the level of the *A. baumannii* complex was carried out using the automated system VITEK 2 (bioMerieux, Inc., Marcy I ’Etoile, France). Colistin susceptibility was assessed by broth microdilution, with resistance described as having a breakpoint of colistin minimum inhibitory concentration (MIC) >2 mg/L [14]. The VITEK 2 system is a completely automated system that uses a fluorogenic technique for identifying species and a turbidimetric approach for measuring susceptibility [15]. CRAB was defined in terms of resistance to carbapenems (imipenem, meropenem), but sensitive to colistin.

### 2.4. Statistical Analysis

Categorical variables were defined as frequencies and percentages, while the mean and standard deviations were reported as continuous variables. Fisher’s exact test for categorical variables was used to compare between two groups, and an independent t-test for continuous variables was used. The results of the two-tailed test with a *p*-value of <0.05 were considered statistically significant. Differences in crude primary outcome rates between the two groups (30-day mortality), and secondary outcomes (clinical response, microbiological response and nephrotoxicity) were compared using Fisher’s exact test for test significance.

Propensity score matching was carried out to decrease possible biases due to imbalances in the baseline characteristics of the treatment groups. The propensity score is calculated using multivariable logistic regression. The variables included in the calculation of the propensity score were gender, hospital length, total colistin dose, septic shock, Charlson score, APACHE II score, colistin treatment courses, baseline glomerular filtration rate (GFR), underlying disease (chronic obstructive pulmonary disease, chronic liver disease, chronic kidney disease, hypertension), sources of CRAB infection (pneumonia, urinary tract infection, other sources of CRAB infections), nephrotoxic medications (vasopressor, amphotericin B, diuretics) and baseline covariates with an inclusion criterion of *p* < 0.25.

Fisher’s exact test was used after matching the propensity score to compare differences in rates of 30-day mortality, clinical response, microbiological response and nephrotoxicity based on colistin monotherapy and colistin plus vancomycin combination therapy.

Furthermore, logistic regression was used to calculate odds ratio (OR) and 95% CI of primary outcome (30-day mortality), and secondary outcomes (clinical response, microbiological response and nephrotoxicity) in patients who received colistin plus vancomycin. Variables with *p* values of 0.20 in the univariate analysis were included in the multivariate analysis, and this was also adjusted (adjusted odds ratio; aOR) for gender, duration of hospitalization, courses of colistin therapy, septic shock, baseline GFR, chronic liver disease, vasopressor, chronic kidney disease, hypertension, pneumonia, urinary tract infection, chronic obstructive pulmonary disease, amphotericin B and diuretics. Statistical significance was defined as a *p*-value of <0.05. All statistical analyses were performed using Stata software, version 14 (Stata-Corp, College Station, TX, USA).

## 3. Results

In total, 365 critically ill patients hospitalized in the ICU with CRAB were included in this study. Of these, 61.37% were females. The study participants were elderly patients with a mean age of approximately 65 years old. The most common underlying diseases were hypertension, cardiovascular disease and diabetes mellitus (Table 1). There were 132 cases (36.16 %) of CRAB treated with colistin alone and 233 cases (63.83%) of CRAB treated with colistin plus vancomycin. The characteristics of study patients and comparisons between colistin alone and colistin plus vancomycin are presented in Table 1.

**Figure 1:**
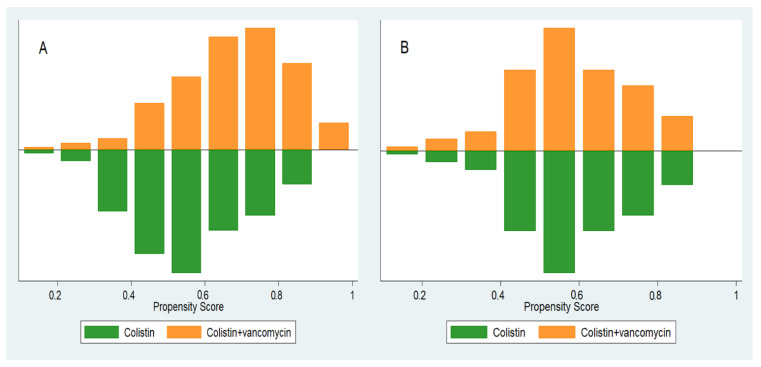
Distribution of propensity scores. (**A**) Propensity score before matching and (**B**) propensity score after matching.

Two hundred and thirty patients were included after matching patients in a 1:1 ratio using propensity score, where 115 were allocated to the colistin monotherapy group and 115 to the colistin plus vancomycin group. With a mean ± SD propensity score of 0.58 ± 0.15, the characteristics of the two groups were identical. Figure 1 shows the distribution of propensity scores between groups before and after matching. The characteristics of the patients between the two groups were largely comparable after propensity matching (Table 1).

### 3.1. Patient Outcomes before Propensity Scoring Match

From a crude comparison of the 30-day mortality rate, it can be determined that 47.73% of patients were in the colistin alone and 51.07% in colistin plus vancomycin groups, respectively, (*p* = 0.586). The rate of clinical response observed was 60.61% of the patients in the colistin alone and 59.23% in colistin plus vancomycin groups, respectively (*p* = 0.825). The rate of microbiological response observed was 67.42% of the patients in the colistin alone and 69.10% in colistin plus vancomycin groups, respectively (*p* = 0.815). Moreover, the rate of nephrotoxicity according to the RIFLE criteria was 49.24% for the colistin monotherapy and 48.93% in colistin plus vancomycin groups, respectively (*p* = 1.000). The analysis for crude outcomes is shown in Table 2.

### 3.2. Patient Outcomes after Propensity Scoring Match

A crude comparison of the 30-day mortality rate shows that 47.83% of patients were in the colistin alone and 47.83% in colistin plus vancomycin groups, respectively, (*p* = 1.000). The rate of clinical response observed was 56.52% of the patients in the colistin alone and 58.26% in colistin plus vancomycin groups, respectively (*p* = 0.894). The rate of microbiological response observed was 65.22% of the patients in the colistin alone and 66.09% in colistin plus vancomycin groups, respectively (*p* = 1.000). However, the rate of nephrotoxicity according to the RIFLE criteria was 51.30% for the colistin monotherapy and 55.65% in colistin plus vancomycin groups, respectively (*p* = 0.634). The analysis for patient outcomes after propensity score matching is shown in Table 2.

### 3.3. Unmatched Cohort Analyses

A logistic regression analysis was adjusted with covariates (in the statistical analysis section) for the primary outcome (30-day mortality) was aOR 0.96, 95% CI, 0.56 to 1.63, (*p* = 0.885) and secondary outcomes (i.e., clinical response, *p* = 0.822; microbiological response, *p* = 0.968; and nephrotoxicity, *p* = 0.785) (Table 3).

### 3.4. Propensity-Matched Cohort Analyses

The results of the propensity score matching analysis using the logistic regression model were similar to those from the unmatched analysis, showing no significant differences in both the primary outcome (30-day mortality) and secondary outcomes (i.e., clinical response, *p* = 0.794; microbiological response, *p* = 0.976; and nephrotoxicity, *p* = 0.474). (Table 3).

## 4. Discussion

In critically ill patients with CRAB infections, 30-day mortality, clinical response and microbiological response do not differ in patients treated with colistin plus vancomycin compared to those receiving colistin alone. Moreover, colistin plus vancomycin therapy had similar nephrotoxicity compared with colistin therapy alone. Based on results from our study, we do not support previous in vitro studies [10,16,17] regarding the potent synergy of colistin plus vancomycin against CRAB infections.

There are multiple knowledge gaps pertaining to the clinical use and utility of colistin in critically ill patients but, due to a lack of options, it is used in these high-risk patients [18]. Until recently, little clinical data have focused on using combinations of colistin and vancomycin against CRAB [18]. Moreover, an acquired antibiotic resistance of CRAB has been recognized as an important therapeutic challenge. Thus, the use of a combination of colistin and vancomycin has recently been reviewed [18].

The rationale for the use of combination therapy against CRAB infection is based on the hypothesis that colistin and vancomycin interact synergistically to increase bacterial killing and produce a combined effect greater than the sum of their separate effects or, conversely, that the same killing effect can be achieved using lower doses of antibiotics [17].

The proposed hypothesis of synergy relates to the outer membrane permeabilization of colistin. Colistin exerts its primary antimicrobial activity by displacing Ca^2+^ and Mg^2+^ ions from the lipid A component of lipopolysaccharide molecules, disrupting the outer membrane of *A. baumannii*. This allows the entry of large and hydrophobic vancomycin molecules, which pass through the outer membrane of *A. baumannii* toward their targets in the cell wall [18].

A previous in vitro study by Gordon et al. [16] first described synergism when colistin was combined with vancomycin against multidrug-resistant (MDR) *A. baumannii*. This was supported by a case series [19] conducted in a pediatric ICU. In order to explore the potential synergistic activity of colistin plus vancomycin, the study found that colistin and vancomycin in combination were highly synergic in four severe cases of pediatric patients caused by MDR *A. baumannii* infections. Colistin and vancomycin combination therapy had a favorable outcome with no infection relapses in four pediatric patients [19]. Consistently, Oliva et al. [20] showed one case of a patient which was successful with the combination colistin plus vancomycin plus rifampin for treatment of ventilator-associated pneumonia (VAP), due to an MDR *A. baumannii* [20]. However, these studies [19,20] had a small sample size and no control group. Furthermore, the antagonism effect of colistin plus vancomycin has been highlighted by in vitro and in vivo studies [21] to evaluate the combination of vancomycin and colistin against MRSA infection, and found that colistin increased the MIC of vancomycin by 0.25 to 0.75 μg/mL. Furthermore, the combination of vancomycin and colistin, showed antagonism in four of five ST5-MRSA strains [21]. Therefore, the combination of colistin and vancomycin has been studied with conflicting results, both with respect to improved outcomes and the risk of nephrotoxicity [22,23].

In our study, we observed that the 30-day mortality (aOR 1.09, 95% CI 0.57–2.08) and clinical response rate (aOR 1.01, 95% CI 0.55–1.84) and microbiological response rate (aOR 1.04, 95% CI 0.56–1.93) showed no significant differences between patients receiving colistin alone and colistin plus vancomycin.

The findings of our study were similar to a retrospective study including episodes of ventilator-associated pneumonia or bacteremia in patients who received colistin alone and colistin plus vancomycin combination therapy against CRAB; the study found that clinical outcomes do not differ in patients treated with colistin plus vancomycin compared to those receiving colistin without vancomycin. However, the rate of acute kidney injury was higher in the combination therapy group (55.2 vs. 28%; *p* = 0.04). However, this study had a small sample size of 57 patients [22].

Moreover, a multi-center retrospective study evaluated the rate of mortality for patients receiving colistin alone and those receiving colistin with a concomitant glycopeptide for treatment of MDR Gram-negative bacteria [23]. Using a sample size of 166 patients with confirmed MDR Gram-negative infections, the 30-day mortality was not significantly different between those treated with the combination and those treated with monotherapy (33.8 versus 29.6%). In addition, nephrotoxicity was not different between patients receiving glycopeptides and those not receiving glycopeptides. This study was performed in a larger population of critically ill Gram-negative bacteria-infected patients, including, though not limited to, CRAB patients [23].

These findings in severely ill patients do not support data on the synergistic activity of colistin plus vancomycin with previous laboratory findings [10,16,17]; neither difference in the 30-day mortality nor difference in clinical response between patients with CRAB infection who were treated with colistin alone or with colistin plus vancomycin were observed in this retrospective study. Several factors might explain these phenomena. Firstly, vancomycin and colistin exhibit poor penetration into the pulmonary parenchyma [24,25]. As the majority of patients in present study suffered from CRAB pneumonia, it may explain the lack of clinical efficacy of this combination. Secondly, synergy testing of colistin and vancomycin against CRAB were not performed given the retrospective nature of the present study. Thirdly, the colistin concentration of 0.5 μg/mL is needed to boost vancomycin activity [17]. However, it was found that patients who received the same loading doses of CBA (300 mg) had different colistin concentrations at a steady state due to a wide range of volume of distribution and clearance of colistin in critically ill patients [26]. So, patients in this study might have different concentrations of colistin, resulting in different bacterial effects.

One of the major concerns regarding the colistin and vancomycin co-administration could be induced nephrotoxicity, but this is still controversial [22,23]. Our study showed no statistically significant difference in nephrotoxicity between groups of patients treated with colistin alone and those treated with colistin in combination with vancomycin (*p* = 0.474). Our results are consistent with Aitullina et al. [11] and Garnacho-Montero [22] found that colistin and vancomycin co-administration are not associated with colistin nephrotoxicity.

There are some limitations in this study. Firstly, we observed a substantial difference between the treatment groups in baseline characteristics, although this difference was also seen in most retrospective studies and was difficult to make similar in both groups, which may have contributed to confounders. For adjusted baseline characteristics, a propensity score-matching approach was used to reduce potential biases. In addition, we performed a multivariate analysis to ensure that in our final multivariate model, statistically significant confounders of clinical plausibility were retained. Secondly, because this was a single-center study, according to local epidemiology, the distribution of genetic resistance mechanisms could vary, which could have affected the impact of combination therapy. Finally, vancomycin serum levels have not been reported, so we have not been able to assess their effect on the outcome and safety.

## 5. Conclusions

There was no significant difference in 30-day mortality between colistin plus vancomycin and colistin alone in critically ill patients with CRAB infections. Moreover, clinical response, microbiological response and nephrotoxicity were not significantly different between colistin plus vancomycin and colistin alone treatment groups. These results suggest that there is no significant difference between colistin treatment alone and in combination with vancomycin according to available data. Thus, the combination of colistin plus vancomycin was not found to be a promising therapy against CRAB infections in critically ill patients.

## Figures and Tables

**Table 1:** Demographic and clinical characteristics of patients who received colistin monotherapy compared to colistin-vancomycin combination therapy.

Characteristic	Unmatched Cohort			Propensity-Matched Cohort		
	Colistin Monotherapy (n = 132)	Colistin-Vancomycin (n = 233)	*p*-Value	Colistin Monotherapy (n = 115)	Colistin-Vancomycin (n = 115)	*p*-Value
Sex, n (%)						
Male	39 (29.55)	102 (43.78)	0.007	37(32.17)	39(33.91)	0.889
Female	93 (70.45)	131 (56.22)		78(67.83)	76(66.09)	
Age, mean + SD (year)	64.20 ± 16.89	65.77 ± 17.71	0.407	64.84 ± 15.95	64.84 ± 17.43	1.000
Duration of treatment, median (IQR)	8.5 (5–13)	10 (7–14)	0.004	9 (5–14)	8 (5–14)	0.542
Underlying disease, n (%)						
• Hypertension	55 (41.67)	114 (48.93)	0.191	48 (41.74)	43 (37.39)	0.590
• Cardiovascular disease	41 (31.06)	83 (35.62)	0.421	36 (31.30)	40 (34.78)	0.674
• Diabetes mellitus	29 (21.97)	54 (23.18)	0.897	27 (23.48)	27 (23.48)	1.000
• Chronic kidney disease	26 (19.70)	65 (27.90)	0.101	25 (21.74)	23 (20.00)	0.871
• Chronic obstructive pulmonary disease	30 (22.73)	36 (15.45)	0.090	22 (19.13)	26 (22.61)	0.627
• Malignancy	29 (22.14)	44 (18.88)	0.518	27 (23.68)	23 (20.00)	0.526
• Chronic liver disease	7 (5.30)	17 (7.30)	0.517	6 (5.22)	11 (9.57)	0.314
Septic shock, n (%)	83 (62.88)	182 (78.11)	0.002	76 (66.09)	77 (66.96)	1.000
Mechanical ventilation, n (%)	117 (88.64)	208 (89.27)	0.863	102 (88.70)	102 (88.70)	1.000
Charlson Score, median (IQR)	2 (0–3)	2 (1–4)	0.206	2 (0–3)	2 (1–4)	0.620
Length of hospital stay, median (IQR) (day)	31.5 (22–48)	39 (25–56)	0.094	32 (22–49)	38 (23–54)	0.154
APACHE II score (mean ± SD)	12.43 ± 4.855	11.68 ± 5.24	0.241	12.53 ± 0.49	11.92 ± 0.59	0.432
Baseline SCr, mg/dL, median (IQR)	0.8 (0.6–1.5)	0.9 (0.6–1.7)	0.647	0.9 (0.6–1.8)	0.9 (0.6–1.5)	0.987
Baseline GFR, mL/min, median (IQR)	71.55 (23.54–104.76)	47.70 (15.03–89.60)	0.007	65.09 (22.37–103.3)	71.44 (28.74–102.57)	0.524
Total colistin dose, median (IQR) (g)	2.10 (1.20–3.00)	1.800 (1.10–3.00)	0.244	1.95 (1.20–3.00)	1.80 (1.10–3.00)	0.517
Type of nephrotoxic medications, n (%)						
• Aminoglycosides	2 (1.52)	4 (1.72)	1.000	2 (1.74)	2 (1.74)	1.000
• Diuretics	100 (75.76)	196 (84.12)	0.053	89 (77.39)	93 (80.87)	0.627
• Amphotericin B	3 (2.27)	22 (9.44)	0.009	3 (2.61)	7 (6.09)	0.333
• Vasopressor	85 (64.39)	182 (78.11)	0.007	78 (67.83)	78 (67.83)	1.000
Sources of CRAB infection						
• Pneumonia	116 (87.88)	203 (87.12)	0.871	101 (87.83)	103 (89.57)	0.835
• Bacteremia	1 (0.76)	2 (0.86)	1.000	1 (0.87)	0 (0.00)	1.000
• UTI	13 (9.85)	14 (6.01)	0.212	12 (10.43)	10 (8.70)	0.823
• Other	11(8.33)	24 (10.30)	0.295	9 (7.83)	10 (8.70)	1.000
Propensity score, mean + SD	0.36 ± 0.17	0.47 ± 0.18	0.006	0.58 ± 0.15	0.58 ± 0.15	0.976

SCr, serum creatinine; GFR, glomerular filtration rate; SD, standard deviation; CRAB, Carbapenem-resistant *Acinetobacter baumannii*; UTI, urinary tract infection; Other, inter costal drainage and surgical site infection; IQR, interquartile range; each patient could have more than 1 drug.

**Table 2:** Crude outcomes, toxicity and mortality rates in patients received colistin monotherapy compared to colistin-vancomycin combination therapy.

Outcome	No. of Patients (%) with Each Outcome with Indicated Treatment		*p*-Value	No. of Patients (%) with Each Outcome with Indicated Treatment		*p*-Value
	Colistin Monotherapy (n = 132)	Colistin-Vancomycin (n = 233)		Colistin Monotherapy (n = 115)	Colistin-Vancomycin (n = 115)	
**Primary outcome**						
30-day mortality rate	63 (47.73)	119 (51.07)	0.586	55 (47.83)	55 (47.83)	1.000
**Secondary outcome**						
Clinical response	80 (60.61)	138 (59.23)	0.825	65 (56.52)	67 (58.26)	0.894
Microbiological response	89 (67.42)	161 (69.10)	0.815	75 (65.22)	76 (66.09)	1.000
Nephrotoxicity	65 (49.24)	114 (48.93)	1.000	59 (51.30)	64 (55.65)	0.634
Risk	22 (16.67)	43 (18.46)		20 (21.51)	28 (27.18)	
Injury	22 (16.67)	34 (14.59)		18 (19.35)	20 (19.42)	
Failure	20 (15.15)	35 (15.02)		20 (21.51)	16 (15.53)	
Loss	1 (0.75)	2 (0.86)		1 (1.08)	0 (0.00)	
ESRD	0 (0.00)	0 (0.00)		0 (0.00)	0 (0.00)	

ESRD, end-stage renal disease.

**Table 3:** Logistic regression analysis of outcomes for critically ill patients receiving colistin monotherapy compared with colistin vancomycin combination therapy.

Variable	Logistic Regression Analysis *		Propensity Score Matched Logistic Regression Analysis *	
	aOR (95% CI)	*p*-Value	aOR (95% CI)	*p*-Value
**Efficacy** **Primary outcome**				
30 days mortality	0.96 (0.56–1.63)	0.885	1.09 (0.57–2.08)	0.794
**Secondary outcomes**				
Clinical response	1.06 (0.63–1.80)	0.822	1.01 (0.55–1.84)	0.976
Microbiological response	1.01 (0.59–1.72)	0.968	1.04 (0.56–1.93)	0.894
**Safety**				
Nephrotoxicity	0.93 (0.57–1.52)	0.785	1.23 (0.70–2.18)	0.474

CI, confidence interval; aOR, adjusted odds ratio. * The multivariate analysis was adjusted for gender, duration of hospital stay, courses of colistin therapy, septic shock, baseline GFR, chronic liver disease, vasopressor, chronic kidney disease, hypertension, pneumonia, urinary tract infection, chronic obstructive pulmonary disease, amphotericin B and diuretics.

## Data Availability

The datasets used and analyzed during the current study are available from the corresponding author on reasonable request.
